# The chromosome-level *Hemerocallis citrina* Borani genome provides new insights into the rutin biosynthesis and the lack of colchicine

**DOI:** 10.1038/s41438-021-00539-6

**Published:** 2021-04-07

**Authors:** Zhixing Qing, Jinghong Liu, Xinxin Yi, Xiubin Liu, Guoan Hu, Jia Lao, Wei He, Zihui Yang, Xiaoyan Zou, Mengshan Sun, Peng Huang, Jianguo Zeng

**Affiliations:** 1 Hunan Key Laboratory of Traditional Chinese Veterinary Medicine, Hunan Agricultural University, Changsha, Hunan 410128 China; 2 College of Veterinary Medicine, Hunan Agricultural University, Changsha, Hunan 410128 China; 3 Wuhan Frasergen Bioinformatics Co., Ltd, Wuhan, Hubei 430075 China; 4 College of Animal Science and Technology, Hunan Agricultural University, Changsha, Hunan 410125 China; 5 Green Melody Bio-engineering Group Company Limited, Changsha, Hunan 410329 China; 6 National and Local Union Engineering Research Center of Veterinary Herbal Medicine Resource and Initiative, Hunan Agricultural University, Changsha, 410128 China

**Keywords:** Comparative genomics, Metabolomics

## Abstract

*Hemerocallis citrina* Borani (huang hua cai in Chinese) is an important horticultural crop whose flower buds are widely consumed as a delicious vegetable in Asia. Here we assembled a high-quality reference genome of *H. citrina* using single-molecule sequencing and Hi-C technologies. The genome assembly was 3.77 Gb and consisted of 3183 contigs with a contig N50 of 2.09 Mb, which were further clustered into 11 pseudochromosomes. A larger portion (3.25 Gb or 86.20%) was annotated as a repetitive content and 54,295 protein-coding genes were annotated in the genome. Genome evolution analysis showed that *H. citrina* experienced a recent whole-genome duplication (WGD) event at ~15.73 million years ago (Mya), which was the main factor leading to many multiple copies of orthologous genes. We used this reference genome to predict 20 genes involved in the rutin biosynthesis pathway. Moreover, our metabolomics data revealed neither colchicine nor its precursors in *H. citrina*, challenging the long-standing belief that this alkaloid causes poisoning by the plant. The results of our disruptive research are further substantiated by our genomic finding that *H. citrina* does not contain any genes involved in colchicine biosynthesis. The high-quality genome lays a solid foundation for genetic research and molecular breeding of *H. citrina*.

## Introduction

*Hemerocallis citrina* Borani is a perennial crop and its flower buds are one of the most commonly consumed vegetables in Asia. This plant has been widely grown in Asian countries, including China, Japan, and Korea, and has also been regarded as the traditional mother’s flower in Chinese culture for a thousand years^1,2^. *H. citrina* flower buds have been used to relieve depression and promote lactation, as documented in the medicinal book “*Compendium of Materia Medica*,” which is a famous Chinese encyclopedia of medicine^3,4^. Modern pharmaceutical studies have demonstrated that *H. citrina* extract has antidepressant, antioxidant, and anti-inflammatory effects^5–7^. The chemical components isolated from *H. citrina* mainly include flavonols, polyphenols, anthraquinones, and alkaloids^8^. Rutin is the main chemical constituent and plays an important role in the antidepressant activity of *H. citrina*^5^; however, the corresponding biosynthetic genes have rarely been reported in this plant. Here we predicted some candidate genes of the rutin biosynthesis pathway by the comparative genomic method. In addition, the relatively fast floral development of *H. citrina* severely restricts the harvest window and places a significant resource strain on post-harvest processing. Moreover, the edible value of *H. citrina* rapidly deteriorates after flowering due to a loss of flavor, leading to substantial food waste. Therefore, it is an urgent task to cultivate new varieties of *H. citrina* with staggered flowering periods or non-blooming buds via molecular breeding, which could generate tremendous economic value. However, the lack of genomic information restricts the cultivation of new varieties and a high-quality genome of *H. citrina* could provide the possibility of achieving this goal.

The market value of *H. citrina* has been ~1 billion US dollars for many years. One of the crucial reasons for the limited market value is that colchicine in the flower buds is widely recognized as a poisonous substance^1^. However, the existence of colchicine in *H. citrina* was questioned by our team several years ago^9^. This study aimed to further determine whether colchicine and its precursors exist in *H. citrina* or not, based on metabolic data, and to clarify why this alkaloid is not produced according to genomic data. The high-quality and chromosome-level genome of *H. citrina* will provide new insights into the rutin biosynthesis and the lack of colchicine.

## Results

### Sequencing and assembly

**Fig. 1:**
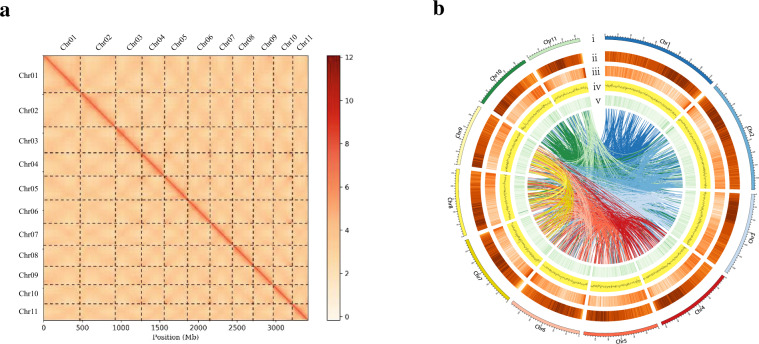
*H. citrina* genome assembly. **a** The Hi-C interaction heatmap for the *H. citrina*. The color in the figure from light to dark indicates the increase in the intensity of interaction. **b** Overview of *H. citrina* genomic features. (i) Circular representation of the pseudomolecule, (ii–v) repetitive sequence density (bin = 1 M), gene density (bin = 1 M), GC content (bin = 1 M), ncRNA densities (bin = 1 M), and genome collinearity

We generated 177.52 Gb of 150 bp paired-end reads and 157.53 Gb (coverage of ~41.46×) of short reads (Supplementary Table S1). Simultaneously, we generated 165-fold PacBio single-molecule long polymerase reads (625.85 Gb with an N50 length of 38.27 kb) and 172-fold Hi-C data (646.63 Gb) were used to construct the chromosome-level high-quality reference genome. The genome size was estimated to be ~3.80 Gb and the heterozygosity rate and repeat sequence contents were 1.28% and 78.85%, respectively (Supplementary Table S2), based on Illumina resequencing data. In the end, we obtained 3183 contigs with an N50 of 2.08 Mb and a size of 3.77 Gb, which was ~99% of the estimated size (Table 1). To construct chromosome-level genes, we used ~170× Hi-C data to anchor contigs to chromosomes. We successfully clustered 2919 contigs spanning 3.41 Gb (90.36% of the total length of all contigs) into 11 chromosome groups after further ordering and orienting the clustered contigs (Fig. 1a). Finally, we obtained the first chromosome-level and high-quality genome of *H. citrina*, with chromosome lengths ranging from 216.66 to 471.57 Mb, accounting for 90.42% of the whole sequence (Fig. 1b and Supplementary Table S3).

**Table 1:** *H. citrina* genome assembly results

	Mecat 2 assembly	Post Gcpp	Post Pilon	Haplotig purge	Hi-C assembly
Size (Mb)	5611.82	5611.82	5611.82	3774.13	3775.58
No. contigs/Scaffold	8877	8877	8877	3183	734
No. contigs/Scaffold (>2 kb)	8834	8834	8842	3174	725
Max. contig/Scaffold length (bp)	21,710,810	21,804,269	21,795,804	21,795,804	471,572,209
Contig/Scaffold N50 size (bp)	1,516,939	1,522,568	1,521,497	2,081,915	294,951,729
Contig/Scaffold N90 size (bp)	428,328	430,376	428,572	761,644	216,659,559
BUSCO	82.78%	—	92.0%	91.5%	91.2%

We first assessed the accuracy and completeness of our assembly results through Benchmarking Universal Single-Copy Orthologs (BUSCO) analysis, then identified 91.4% complete and 2.4% partial BUSCO genes (Supplementary Table S4). In addition, 99.49% of the filtered short reads (157.53 Gb, Supplementary Table S1) were mapped to the genome of *H. citrina*, which covered 99.86% of the assembly. Furthermore, a total of 22,310 homozygous single-nucleotide polymorphisms (SNPs) (0.0006% of the total *H. citrina* assembly) were identified. In summary, the above results demonstrate the high accuracy and completeness of the *H. citrina* genome.

### Genome annotation

Repetitive sequence prediction of the *H. citrina* genome was mainly performed through two methods: homology annotation and ab initio prediction. A total of 3.25 Gb of repetitive elements was identified in our assembled genome, comprising 86.20% of the whole genome (Supplementary Table S5). Among these repetitive elements, long terminal repeats were the main type, accounting for 72.39% (2.73 Gb). The rest were short interspersed nuclear elements, DNA transposons, and long interspersed nuclear elements, which accounted for 0.15%, 14.24%, and 6.63%, respectively. Similarly, a total of 3540 transfer RNA (tRNA), 406 ribosomal RNA, 457 small nuclear RNA, and 127 microRNA genes were annotated in the *H. citrina* genome (Supplementary Table S6).

We predicted 54,295 protein-coding genes in the *H. citrina* genome, with an average length of 8339 bp and an average exon number of 4.53 for each gene (Supplementary Table S7). By comparing the genes annotated in the other six species, we found that the various indicators of the annotated genes (gene, CDS, exon, and intron lengths) were similar to those of other species (Supplementary Fig. S1). We functionally annotated ~44,398 (81.77%) protein-coding genes of *H. citrina* based on known genes, conserved domains, and Gene Ontology (GO) terms (Supplementary Table S8). Finally, 93.8% of the BUSCO genes were identified in the annotation of *H. citrina* (Supplementary Table S4), which showed that our annotations were complete and reliable by BUSCO analysis.

### Genome evolution and gene families expansion/contraction

**Fig. 2:**
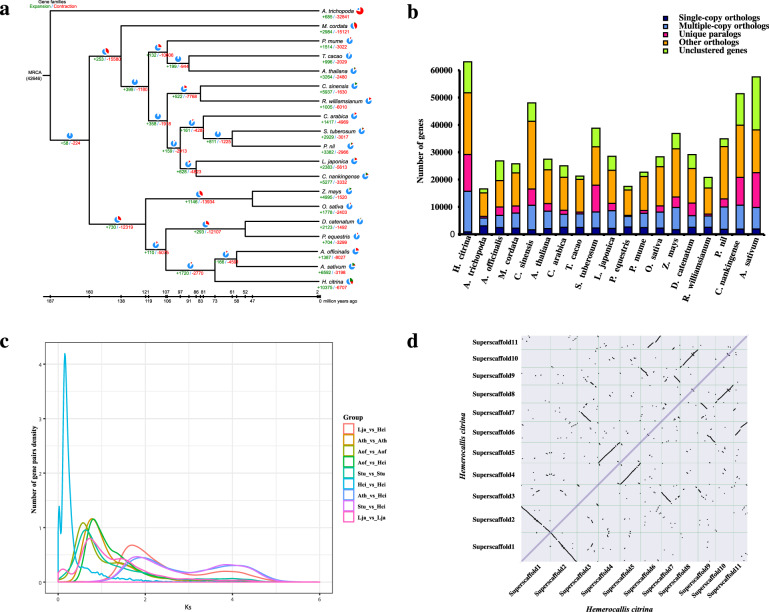
Evolution of the *H. citrina* genome and gene families. **a** Phylogenetic tree of 19 plant species and the sizes of expanded and contracted gene families: *A. trichopoda*, *M. cordata*, *P. mume*, *T. cacao*, *A. thaliana*, *C. sinensis*, *R. williamsianum*, *C. arabica*, *S. tuberosum*, *P. nil*, *L. japonica*, *C. nankingense*, *Z. mays*, *O. sativa*, *D. catenatum*, *P. equestris*, *A. officinalis*, *A. sativum*, and *H. citrina*. **b** The distribution of single-copy, multiple-copy, unique, other orthologous and unclustered genes in the 19 plant species. **c** Distribution of Ks values between *L. japonica*, *A. thaliana*, *A. officinalis*, *S. tuberosum*, and *H. citrina* (Ks distribution chart showing that *H. citrina* experienced a relatively recent WGD event). **d** Dot plots of paralogs in the *H. citrina* genome

In this study, we first compared the protein sequences encoded by *H. citrina* with those encoded by 18 other species, namely, *Amborella trichopoda*, *Macleaya cordata*, *Prunus mume*, *Arabidopsis thaliana*, *Theobroma cacao*, *Camellia sinensis*, *Rhododendron williamsianum*, *Solanum tuberosum*, *Pharbitis nil*, *Coffee arabica*, *Chrysanthemum nankingense*, *Lonicera japonica*, *Dendrobium catenatum*, *Phalaenopsis equestris*, *Asparagus officinalis*, *Allium sativum*, *Oryza sativa*, and *Zea mays*. These species had 116 single-copy orthologous gene families according to gene family cluster analysis. In addition, we clustered 51,740 protein sequences (81.99%) encoded by *H. citrina* into 15,974 gene families. After length-based filtering of the shared single-copy orthologous gene families, 116 genes remained. The phylogenetic tree showed that the *H. citrina*, *A. sativum*, and *A. officinalis* were located on the same evolutionary branch, showed a closer relationship. In addition, our prediction results showed that *H. citrina*, *A. sativum*, and *A. officinalis* phylogenetically diverged from the common ancestor ~71.7 Mya, after the separation of Orchidaceae at 107.24 Mya (Fig. 2a), which is consistent with published research^10^.

A total of 42,646 gene families in the most recent common ancestor of the 19 species were obtained by analyzing the gene family expansion and contraction. The number of expanded and contracted gene families in *H. citrina* were 10,375 and 6707, respectively (Fig. 2a). Compared with *A. officinalis* and *A. sativum*, it has 116 expanded and 4591 contracted gene families, which demonstrated that the number of expanded genes in *H. citrina* had increased significantly. This result indicated that *H. citrina* may have experienced more duplication events than *A. officinalis* and *A. sativum*. We found that these genes in *H. citrina* were also the most abundant based on the multicopy homologous genes number (Fig. 2b). In addition, we performed GO and KEGG (Kyoto Encyclopedia of Genes and Genomes) enrichment analyses of these expanded and contracted genes in the *H. citrina* genome. We found lineage-specific expansions of genes related to the metabolic biosynthesis of flavonoids, which may affect the biosynthesis of rutin and enhance flavor and medicinal value (Supplementary Table S9).

### Genome-wide duplication events

To identify the source of many genes (>50,000) in *H. citrina*, we performed whole-genome duplication (WGD) analysis using overlapping *H. citrina* genomes. The synonymous substitution rate (Ks) estimates were applied to detect WGD events. The distribution of Ks values results showed that *H. citrina* have one main peak at Ks values of ~0.18 (~15.73 Mya) (Fig. 2c), whereas *A. officinalis* have more ancient WGD event. Dot plots can be shown as paralogs (2–2 diagonal relationships) evolving from a recent WGD event in the *H. citrina* genome (Fig. 2d).

### Prediction of rutin biosynthesis genes in *H. citrina*

**Fig. 3:**
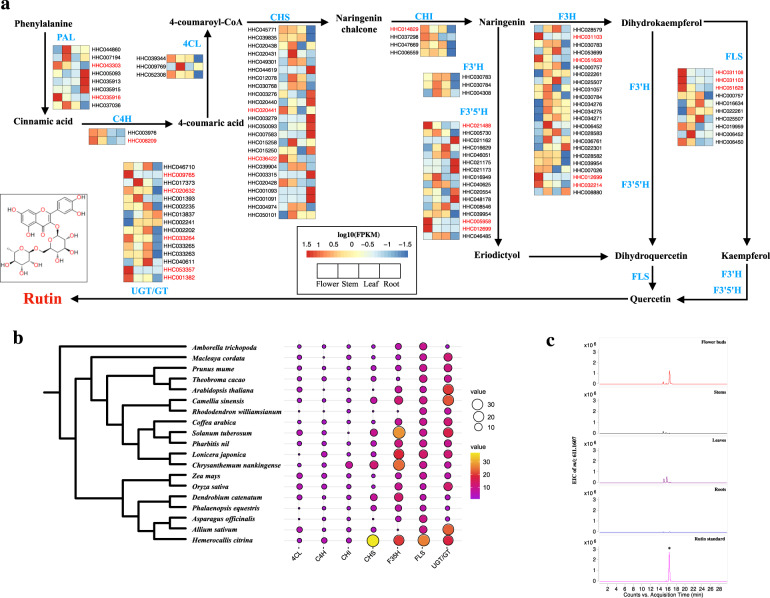
Prediction of rutin biosynthesis genes in *H. citrina.* **a** The biosynthesis pathway and the expression profile for rutin in four tissues: flowers, stems, leaves, and roots. Expression profiles of 108 candidate enzymes are illustrated using a gradient color. Gene expression values (FPKM) were scaled by log10 and the genes with an expected expression pattern were indicated in red. **b** The seven gene families are expanding and contracting, and the heat map mainly shows the number of genes in the *4CL*, *C4H*, *CHS*, *CHI*, *F3’5’H*, *FLS*, and *UGT/GT* gene families. **c** EIC of *m*/*z* value of rutin (*m*/*z* 611.1607, [M + H]^+^) in the TICs of different tissues of *H. citrina*

Rutin is the main ingredient and is the recognized one of the main antidepressant compounds in *H. citrina*. The biosynthetic precursor of rutin is derived from phenylalanine and then synthesized by ten enzymes^11^ (Supplementary Table S10 and Fig. 3a). Through analysis, the expanding and contracting of seven gene families involved in the biosynthesis of rutin. We found four homologous genes (*CHS*, *F3’5’H*, *FLS*, and *UGT/GT*) in *H. citrina* have been increased significantly compared with other species (Fig. 3b). We predicted 108 candidate genes in ten gene families involved in rutin biosynthesis by homologous alignment and a Pfam database search (Fig. 3a). Then, we found that rutin primarily accumulates in the flower buds, whereas the content of rutin in the stems, leaves, and roots is lower according to High-performance liquid chromatography/quadrupole time-of-flight (HPLC-Q-TOF) methods (Fig. 3c), which indicated that the candidate genes were mainly expressed in flower buds. Finally, 20 candidate genes were predicted in line with this coexpression pattern (Fig. 3a, red color).

### Colchicine and its biosynthesis pathway is not existent in *H. citrina*

**Fig. 4:**
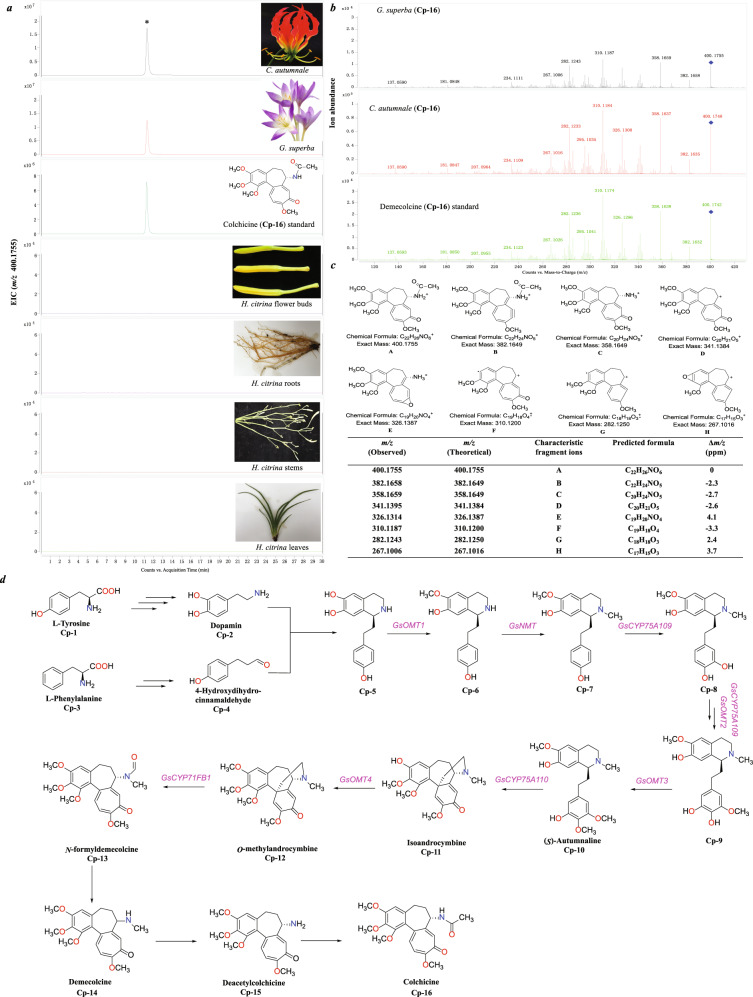
Detection and identification of colchicine. **a** EIC of the theoretical *m*/*z* value (400.1755, [M + H]^+^) of colchicine in the TICs of *G. superba*, *C. autumnale*, *H. citrina*, and the standard. **b** MS/MS spectra of the parent ions at *m*/*z* 400.1755 and 400.1748 from the TICs of *G. superba* and *C. autumnale*, respectively, compared to the colchicine standard (Cp **16**, *m*/*z* 400.1742). **c** The putative structures and tabulated list for the fragment ions from the MS/MS analysis of the mother ion at *m*/*z* 400.1755. **d** Near-complete biosynthesis pathway of colchicine and related functional genes

The extracted ion chromatogram (EIC) of the precise *m*/*z* value of colchicine standard (Cp **16**, *m*/*z* 400.1074, [M + H]^+^) for the total ion chromatograms (TICs) of *Gloriosa superba*, *Colchicum autumnale*, and *H. citrina* were performed. Colchicine was found and identified unambiguously in *G. superba* and *C. autumnale* by comparing their retention time, precise *m*/*z* value, and characteristic fragment ions with those of the standard. However, the precise *m*/*z* value of this compound was not found in the TICs of different tissues of *H. citrina* (Fig. 4a–c), which proved that this plant did not contain colchicine.

The near-complete biosynthesis pathway and related functional genes of colchicine have been identified in *G. superba*^12^ (Fig. 4d). The EIC of the theoretical *m*/*z* values of 15 compounds (Cp **1** to Cp **15**), which were the precursors of colchicine, were performed for the TICs of *G. superba*, *C. autumnale*, and *H. citrina*. These precursors were detected and determined from *G. superba* and *C. autumnale* by their precise *m*/*z* values and characteristic fragment ions. However, only two original amino acids, l-tyrosine (Cp **1**) and l-phenylalanine (Cp **3**), were observed and identified in *H. citrina* (Supplementary Figs. S2 and S4), and the theoretical *m*/*z* values of the remaining 13 precursors were not found (Supplementary Figs. S3 and S5–S16). In addition, the candidate genes involved in the colchicine biosynthesis pathways were detected by BLASTP searching with eight known genes from *G. superba*^12^ (with an *E*-value ≤ 1e – 5, a coverage ≥ 0.5, and an identity ≥ 0.5) (Fig. 4d and Supplementary Table S11). Unsurprisingly, none of the orthologous genes were obtained from *H. citrina*. Therefore, the genomic analysis demonstrated that *H. citrina* does not contain any genes involved in colchicine biosynthesis.

## Discussion

We construct a high-quality and chromosome-level reference genome by combining PacBio SMRT and Hi-C technology. We found that the genome data of *H. citrina* have high heterozygosity and repetitive content features. More importantly, we can use the genome to research the phylogenetic and evolutionary characteristics at a deeper level, and to cultivate new varieties of *H. citrina* with staggered flowering periods or non-blooming buds via molecular breeding. Based on the multi-omics analysis, we deduced a gene coexpression rule and predicted that 20 candidate genes match this rule. These results lay the groundwork for further research on the functional genes involved in the biosynthesis pathway of rutin.

Numerous journals, magazines, newspapers, and other news outlets have reported that *H. citrina* contains colchicine, which was first identified in *Hemerocallis* by microchemical methods in 1929^13^; however, the identification method and result were doubted by other scientists in 1949^14^. In 1977, colchicine was first reported from *H. citrina* in China^15^. In the next few decades, more than 30 poisoning incidents were recorded in China due to the consumption of the fresh flower buds of *H. citrina*, which resulted to more than 830 people with symptoms of poisoning. All reports stated that the poisoning was caused by colchicine in *H. citrina* (Supplementary Table S12). Moreover, *H. citrina* containing colchicine was even recorded in college textbooks and popular science books in China^16–19^. However, this compound was not found in different tissues of *H. citrina* using HPLC-Q-TOF-mass spectrometry (MS) technologies in this study. In addition, none of the orthologous genes involved in the colchicine biosynthesis pathway were identified in the *H. citrina* genome, which further clarified that this alkaloid was absent at the genomic level. Both results unambiguously demonstrate that *H. citrina* does not contain colchicine. In past studies, colchicine was never isolated and identified from *H. citrina* by phytochemical methods. In addition, this alkaloid was only determined by thin-layer chromatography or HPLC by comparing the Rf value or the retention time (Rt) with that of the standard^20,21^, so another compound (*m*/*z* 455.1455 in positive mode) had Rf and Rt values close to those of colchicine (*m*/*z* 400.1755 in positive mode). Therefore, this compound was incorrectly identified as colchicine^9^. This study challenges the long-standing belief that colchicine present in *H. citrina* leads to poisoning.

## Conclusion

Here, a high-quality and chromosome-scale *H. citrina* genome was reported. The genome was ~3.8 Gb in size, with a heterozygosity rate of ~1.28% and contig N50 of 2.09 Mb. Subsequently, Hi-C technology was applied and we anchored 90.42% of the assembled contigs to 11 pseudochromosomes. We identified a total of 54,295 protein-coding genes and 63,105 transcripts. Based on comparative genomics, we found that *H. citrina* experienced a recent WGD event at ~15.73 Mya that increased the number of genes by more than 50 thousand and expanded gene families by more than 10 thousand. A total of 4 gene families involved in the rutin biosynthesis pathway were expanded and 20 candidate genes were predicted by multi-omic data. Finally, we proved for the first time that the biosynthesis pathway of colchicine does not exist in the genome of *H. citrina*. Our research provided the first chromosome-level genome of the *Hemerocallis* genus, which laid the foundation for genetic research and molecular breeding of *H. citrina*.

## Materials and methods

### Sample collection and high-throughput sequencing

The *H. citrina* was cultivated at Hunan Agricultural University. We collected the healthy leaves from the best-growing *H. citrina*. A modified cetyltrimethyl ammonium bromide (CTAB) method^22^ was used for DNA extraction. RNA contaminants were removed by RNase A and the integrity of DNA was obtained. The DNA molecules were used to construct a library after being cut into ~30 kb fragments and then sequenced on the PacBio Sequel II platform (Frasergen, China). Simultaneously, a library with an insert size of 350 bp was constructed for the Illumina HiSeq X Ten platform (Illumina, Inc., San Diego, CA, USA). These short reads for whole-genome sequencing were mainly used for genome survey, error correction, and polishing after initial assembly. A Hi-C library was established using the young leaves of *H. citrina* and the BGI MGISEQ-2000 platform (BGI, China) was used for sequencing. In addition, the size of *H. citrina* genome was evaluated by *k-mer* analysis with GCE^23^ (Supplementary Fig. S17).

### RNA extraction and Iso-Seq sequencing

*H. citrina* was grown in Qidong County (Hunan, China, coordinates: 111°52′22.44″E, 26°53′23.75″N) for RNA extraction. We sampled fresh, healthy roots, stems, leaves, and flowers from five different periods with three biological duplication. We used TRIzol reagent (Invitrogen, USA) to extract total RNA based on the recommended protocol. DNA was removed via RQ1 DNase (Promega, USA). Finally, RNA from all samples was mixed to construct the library.

The cDNA synthesis kit (ClontechSMARTer®) was used to establish the cDNA libraries. AMPure PB beads were employed for the cDNA product purification. A total of 376.06 Mb was sequenced with 30 h movies by PacBio Sequel II platform (Supplementary Table S1). Simultaneously, these RNAs were used to construct short-fragment libraries and then processed on the BGI platform, which yielded 30.74 Gb of raw RNA sequence data with a read quality Q30 of 91.0%.

### Genome assembly

All subread data from SMRT sequencing were used for *H. citrina* genome assembly. The draft genome assembly was obtained using mecat 2 (20,190,226) with the default parameters. The gcpp in the SMRT link 4 toolkit was performed to correct errors after the initial assembly of the genome. Then, we used 157.53 Gb of short reads to correct any remaining errors with Pilon^24^ (v1.22). Due to the heterozygosity of the genome, Haplotigs purge was used to filter redundant sequences^25^.

Pseudochromosomes were determined using Hi-C analysis, as described previously^26^. Briefly, 646.63 Gb of clean read pairs were produced from the Hi-C library and mapped to the polished *H. citrina* contig assembly using BWA (bwa-0.7.17) with the default parameters^27^. LACHESIS^28^ tool was used to cluster contigs into chromosome-level scaffolds by the genomic proximity signal of Hi-C data.

### Evaluation of genome quality

Genome assembly accuracy and completeness were first assessed using the continuous long reads subreads. A total of 96.60% of subreads were mapped to 99.97% of the genome, with an average depth of 129.89×. Then, a window of size 10 kb was used to continuously slide along the genome without overlapping (when the sequence length was <10 kb, the actual length prevailed), calculate the average sequencing depth of the sequence in the window and the percentage of GC content. Finally, draw the contig GC content distribution-sequencing depth distribution density map based on the statistical data (Supplementary Fig. S18). Second, the single-base level genome assembly was evaluated using Illumina short-read by BWA 0.7.17 software^27^. Furthermore, homozygous SNPs were filtered by the GATK 4.0.8.1^29^ package. The assembled genome was also subjected to BUSCO v3.0.2^30^ analysis with embryophyta_odb10 to evaluate the completeness of the genome and annotation.

### Annotation of repetitive sequences and genes

De novo and homology-based prediction methods were employed to annotate the repeat sequences in the genome of *H. citrina*. The known transposable elements within the *H. citrina* genome were identified by combining RepeatMasker^31^, RepeatProteinMask, and RepeatModeler. In addition, the tRNA-related genes were mainly identified by tRNAscan-SE (v1.3.1)^32^ and Infernal (v1.1.2)^33^ software with default parameters.

The assembled genome of *H. citrina* was hard and soft masked by RepeatMasker prior to gene prediction. First, we used homologous proteins to train the gene models of Augustus (v3.3.1)^34^ and SNAP^35^, and then performed ab initio gene prediction based on these models. Second, the protein sequences were predicted genes using Exonerate (v2.2.0)^36^ with the default parameters. Third, the clean RNA-Sequencing reads were assembled into transcripts via Trinity^37^ to perform RNA-based gene prediction and the gene structure was further predicted using PASA^38^. Finally, Maker (v3.00)^39^ was employed to integrate the prediction results of the three strategies.

Gene functions were inferred by aligning our annotated gene models with known databases. BLAST+ (v2.6.0+)^40^ was performed against the National Center for Biotechnology Information (NCBI), Non-Redundant, TrEMBL, and Swiss-Prot^41^. The protein domains were annotated using PfamScan^42^ and InterProScan (v5.35–74.0)^43^ based on InterPro protein databases. The motifs and domains were identified by Pfam^44^. GO^45^ IDs for each gene were obtained from Blast2GO^46^. KEGG Automatic Annotation Server was used to annotate the KEGG pathways^47^.

### Gene family identification

To cluster families of protein-coding genes, proteins from the longest transcripts of each gene from *H. citrina* and other closely related species, including *A. trichopoda*, *M. cordata*, *P. mume*, *A. thaliana*, *T. cacao*, *C. sinensis*, *R. williamsianum*, *S. tuberosum*, *P. nil*, *C. arabica*, *C. nankingense*, *L. japonica*, *D. catenatum*, *P. equestris*, *A. officinalis*, *A. sativum*, *O. sativa*, and *Z. mays*, were used. All proteins were extracted and aligned with each other using BLASTP^40^ programs (NCBI blast v2.6.0) with a maximal *E*-value of 1*e* − 5. We filtered out and excluded putative fragmented genes with an identity <30%, a coverage <50%, and protein-encoding sequences shorter than 50 bp. Then, we used OrthoMCL (v14–137)^48^ to cluster genes from different species into gene families.

### Phylogenetic analysis

We construct a phylogenetic tree of *H. citrina* and other closely related species by the protein sequences of 186 single-copy orthologous genes, which were aligned with the MUSCLE (v3.8.31)^49^ program, and we further employed RAxML (v8.2.11)^50^ to build the phylogenetic tree.

### Gene families expansion/contraction

According to the identified gene families and the constructed phylogenetic tree with the predicted divergence times of those species, we used CAFÉ^51^ to analyze gene families expansion and contraction. Families with a *p*-value < 0.05 were considered to have an accelerated rate of gene gain or loss. These gene families in *H. citrina* (*p*-value ≤ 0.05) were mapped to KEGG pathways for functional enrichment analysis, which was conducted using enrichment methods. For this process, hypergeometric test algorithms were implemented and the *Q*-value (false discovery rate) was calculated to adjust *p*-values utilizing the R environment (https://github.com/StoreyLab/qvalue).

### Whole-genome duplication analysis

We used the synonymous substitution rate (Ks) to detect WGD events. First, syntenic paralogous blocks were identified with MCSCAN between *L. japonica*, *A. thaliana*, *A. officinalis*, *S. tuberosum*, and *H. citrina*. Then, the protein sequences of these plants in the syntenic paralogous blocks were aligned against each other with Blastp (*E*-value ≤ 1*e* − 5) to identify the conserved paralogs of each plant. Third, the Ks values of these gene pairs were calculated. Finally, the Ks distribution was used to evaluate the WGD events.

### Sample collection and preparation for metabolomic analysis

*G. superba*, *C. autumnale*, and *H. citrina* plants were collected from Kunming University of Science and Technology, China Pharmaceutical University, and Hunan Agriculture University, respectively. All samples (whole *G. superba* and *C. autumnale*, and flower buds, roots, stems, and leaves of *H. citrina*) were freeze-dried and crushed by a disintegrator. Approximately 0.4 g of powdered sample was extracted using ultrasonic bath for 120 min with 10 mL of 70% methanol-water (v/v). The extract solution was filtered by a 0.22 μm microporous membrane and stored in a bottle.

### HPLC-Q-TOF-MS conditions

HPLC-Q-TOF-MS conditions were optimized based on the previous method^1^. The gradient of elution was modified as follows: 0–3 min, 10–15% (B); 3–8 min, 15–30% (B); 8–16 min, 30–65% (B); and 16–30 min, 65–95% (B). The injection volume was reduced to 2 μL and the MS/MS data of each compound were obtained using different collision energy (10–35 eV).

## Supplementary information

Supporting Material

Supporting Material

## Data Availability

All sequencing data were deposited in the NCBI Sequence Read Archive (SRA) database with BioProject accession number PRJNA647253. The assembled genome was submitted to DDBJ/ENA/GenBank with accession number JACEHZ000000000. The version is JACEHZ010000000.
