# Supplementary material for: The chromosome-level Hemerocallis citrina Borani genome provides new insights into the rutin biosynthesis and the lack of colchicine
Source: Hortic Res. 2021 Apr 7;8:89. doi: 10.1038/s41438-021-00539-6 (PMC8027641; doi:10.1038/s41438-021-00539-6)

**The chromosome-level *Hemerocallis citrina* Borani genome provides new insights into the rutin biosynthesis and the lack of colchicine**

Table S1 Summary of sequencing data generated in this study.

| Library type | Platform | Read length | Clean reads | Clean base | Coverage | Application |
| --- | --- | --- | --- | --- | --- | --- |
| Long reads | PacBio Sequel | 25,059bp (N50) | 34,789,148 | 625.40Gb | 164.58 x | Genome assembly |
| Short reads | Illumina | 2 x 150bp | 2 x 534,516,958 | 157.53Gb | 41.46 x | Genome survey and base level correction |
| Hi-C | BGI | 2 x 150bp | 2 x 2,155,423,807 | 646.63Gb | 171.52 x | Chromosome construction |
| Iso-Seq | PacBio Sequel | 1,816 bp (N50) | 247,279 | 376.06Mb | - | Genome annotation |
| RNA-Seq | BGI | 2 x 150 | 2 x 100,133,400 | 29.81Gb | - | Genome annotation |

Table S2. Summary of the *K-mer* analysis results of *H. citrina*.

| Sample | *K-mer* number | K-mer Depth | Genome Size (Gb) | Heterozygous Ratio (%) | Repeat (%) |
| --- | --- | --- | --- | --- | --- |
| *H. citrina* | 140,415,959,372 | 35 | 3.80 | 1.28 | 78.85 |

Table S3 Summary of the assembled *H. citrina* pseudo-chromosomes.

| Superscaffold | Number of Contigs | Length of Contigs | Length of Superscaffold |
| --- | --- | --- | --- |
| Superscaffold 1 | 361 | 471,393,207 | 471,572,209 |
| Superscaffold 2 | 362 | 446,061,471 | 446,241,971 |
| Superscaffold 3 | 273 | 331,277,553 | 331,413,054 |
| Superscaffold 4 | 280 | 312,014,283 | 312,153,783 |
| Superscaffold 5 | 265 | 297,432,130 | 297,564,130 |
| Superscaffold 6 | 257 | 294,823,729 | 294,951,729 |
| Superscaffold 7 | 242 | 291,316,855 | 291,437,355 |
| Superscaffold 8 | 218 | 267,149,822 | 267,257,823 |
| Superscaffold 9 | 224 | 245,534,525 | 245,645,526 |
| Superscaffold 10 | 245 | 239,066,599 | 239,188,100 |
| Superscaffold 11 | 192 | 216,564,558 | 216,659,559 |
| TOTAL | 2,919 | 3,412,634,732 | 3,414,085,239 |

Table S4. BUSCO assessment results

|  | Assembly | | Annotation | |
| --- | --- | --- | --- | --- |
|  | Proteins | Percentage (%) | Proteins | Percentage (%) |
| Complete BUSCOs | 1,474 | 91.4 | 1,489 | 92.3 |
| Complete and single-copy BUSCOs | 1,068 | 66.2 | 1,008 | 62.5 |
| Complete and duplicated BUSCOs | 406 | 25.2 | 481 | 29.8 |
| Fragmented BUSCOs | 39 | 2.4 | 51 | 3.2 |
| Missing BUSCOs | 101 | 6.2 | 74 | 4.5 |
| Total BUSCO groups searched | 1,614 | 100.0 | 1,614 | 100.0 |

Table S5. The statistics of classification results of repeated sequences

|  | RepeatMasker TEs | RepeatProteinMask TEs | *De novo* | Combined TEs | |
| --- | --- | --- | --- | --- | --- |
| Type | Length (bp) | Length (bp) | Length (bp) | Length (bp) | % in Genome |
| DNA | 87,592,444 | 4,731,260 | 484,481,769 | 537,569,218 | 14.24 |
| LINE | 61,442,883 | 3,441,991 | 225,915,785 | 250,489,450 | 6.63 |
| SINE | 201,325 | 0 | 5,372,168 | 5,572,896 | 0.15 |
| LTR | 484,148,892 | 559,231,601 | 2,681,703,958 | 2,732,977,094 | 72.39 |
| Other | 71,159 | 0 | 89,374 | 160,533 | 0.00 |
| Unknown | 432,463 | 0 | 65,350,810 | 65,777,388 | 1.74 |
| Total TE | 621,722,650 | 567,404,240 | 3,183,124,242 | 3,254,690,120 | 86.20 |

Table S6. Statistics of non-coding RNA annotation results

| Type | | Copy | Average length(bp) | Total length(bp) | % of  genome |
| --- | --- | --- | --- | --- | --- |
| miRNA | | 127 | 123.22 | 15,649 | 0.0004 |
| tRNA | | 3,540 | 75.45 | 267,107 | 0.0071 |
| rRNA | rRNA | 406 | 266.52 | 108,206 | 0.0029 |
|  | 18S | 49 | 1,248.12 | 61,158 | 0.0016 |
|  | 28S | 46 | 197.28 | 9,075 | 0.0002 |
|  | 5.8S | 27 | 166.67 | 4,500 | 0.0001 |
|  | 5S | 284 | 117.86 | 33,473 | 0.0009 |
|  | 8S | 0 | 0 | 0 | 0 |
| snRNA | snRNA | 457 | 131.48 | 60,085 | 0.0016 |
|  | CD-box | 196 | 104.15 | 20,413 | 0.0005 |
|  | HACA-box | 5 | 125.6 | 628 | 0 |
|  | splicing | 256 | 152.52 | 39,044 | 0.001 |
|  | scaRNA | 0 | 0 | 0 | 0 |

**Table S7. The statistical results of gene prediction**

| Gene set | | Number | Average gene length (bp) | Average CDS length (bp) | Average exon per gene | Average exon length (bp) | Average intron length (bp) |
| --- | --- | --- | --- | --- | --- | --- | --- |
| *De novo* | AUGUSTUS | 46,867 | 11,040.37 | 1,013.17 | 4.36 | 232.23 | 2,981.79 |
|  | SNAP | 91,564 | 38,237.09 | 652.63 | 4.90 | 133.11 | 9,630.05 |
| Homo  log | *S.tuberosum* | 52,692 | 5,334.26 | 728.74 | 3.13 | 233.03 | 2,165.05 |
|  | *O.sativa* | 79,074 | 9,728.57 | 871.07 | 2.96 | 294.38 | 4,521.56 |
|  | *A.thaliana* | 47,500 | 6,468.67 | 820.51 | 3.76 | 218.44 | 2,049.21 |
|  | *L.japonica* | 48,899 | 6,607.37 | 802.54 | 3.58 | 223.94 | 2,246.66 |
|  | *A.setaceus* | 59,524 | 6,948.81 | 818.90 | 3.70 | 221.56 | 2,273.63 |
|  | *M.cordata* | 54,964 | 6,843.82 | 800.74 | 3.56 | 224.85 | 2,359.39 |
| trans.orf/RNAseq | | 1,135,527 | 816.95 | 415.46 | 1.43 | 290.86 | 938.23 |
| trans.orf/ISOseq | | 20,612 | 13,739.08 | 1,166.18 | 6.38 | 293.48 | 2,204.54 |
| MAKER | | 54,295 | 8,339.42 | 938.12 | 4.53 | 269.66 | 2,015.65 |

Table S8. The statistical of functional annotations

| Type | | Number | Percent (%) |
| --- | --- | --- | --- |
| Total | | 54,295 | 100.00 |
| Annotated | InterPro | 33,939 | 62.51 |
|  | GO | 23,125 | 42.59 |
|  | KEGG_ALL | 43,685 | 80.46 |
|  | KEGG_KO | 17,697 | 32.59 |
|  | Swissprot | 32,742 | 60.30 |
|  | TrEMBL | 43,664 | 80.42 |
|  | NR | 44,202 | 81.41 |
| Annotated | | 44,398 | 81.77 |
| Unannotated | | 9,897 | 18.23 |

Table S10. The 10 enzymes involved in the biosynthesis pathway of rutin

| Number | Enzymes | Abbreviation |
| --- | --- | --- |
| 1 | Phenylalanine ammonia-lyase | *PAL* |
| 2 | Cinnamate-4-hydroxylase | *C4H* |
| 3 | 4-coumarate CoA ligase | *4CL* |
| 4 | Chalcone synthase | *CHS* |
| 5 | Chalcone isomerase | *CHI* |
| 6 | Flavanone-3’-hydroxylase | *F3’H* |
| 7 | Flavanone-3’-5’-hydroxylase | *F3’5’H* |
| 8 | Flavanone-3-hydroxylase | *F3H* |
| 9 | Flavonol synthase | *FLS* |
| 10 | Glucosyl/rhamnosyl transferase | *UGT/GT* |

Table S11. The 8 identified biosynthetic genes from *G. superba*

| Gene name | NCBI Genbank number |
| --- | --- |
| *GsOMT1* | MT512039 |
| *GsNMT* | MT512040 |
| *GsCYP75A109* | MT512042 |
| *GsOMT2* | MT512043 |
| *GsOMT3* | MT512044 |
| *GsCYP75A110* | MT512045 |
| *GsOMT4* | MT512046 |
| *GsCYP71FB1* | MT512047 |

**Table S12 The poisoning events recorded in China since 1978**

| NO. | Years | Location | Num. of poisoning | References |
| --- | --- | --- | --- | --- |
| 1 | 1978 | Kunming,Yunnan province | 42 | [1] |
| 2 | 1979 | Engineering Corps | 94 | [2] |
| 3 | 1988 | Xiangxiang City, Hunan Province | 27 | [3] |
| 4 | 1991 | 36175 troops | 42 | [4] |
| 5 | 1993 | / | 21 | [5] |
| 6 | 1996 | Qingdao City, Shandong Province | 23 | [6] |
| 7 | 1996 | Shantou City, Guangdong Province | 35 | [7] |
| 8 | 1998 | Xunke County, Heilongjiang Province | 2 | [8] |
| 9 | 1998 | Zibo City, Shandong Province | 22 | [9] |
| 10 | 1999 | Qingdao City, Shandong Province | 90 | [10] |
| 11 | 1999 | Weifang City, Shandong Province | 15 | [11] |
| 12 | 1999 | Qixia City, Shandong Province | 60 | [12] |
| 13 | 2000 | Qingdao City, Shandong Province | 3 | [13] |
| 14 | 2000 | Huian County, Fujian Province | 38 | [14] |
| 15 | 2000 | Fengtai County, Anhui Province | 8 | [15] |
| 16 | 2000 | Cao country, Heze city, Shandong province | 4 | [16] |
| 17 | 2001 | Changzhi County, Shanxi Province | 2 | [17] |
| 18 | 2002 | Fuxin County, Liaoning Province | 2 | [18] |
| 19 | 2003 | Liuzhou City, Guangxi Province | 34 | [19] |
| 20 | 2003 | Liuzhou City, Guangxi Province | 34 | [20] |
| 21 | 2003 | Qingyang District, Chengdu | 28 | [21] |
| 22 | 2003 | Beijing | 28 | [22] |
| 23 | 2004 | Hedong District, Tianjin | 15 | [23] |
| 24 | 2004 | Fushun, Liaoning province | 18 | [24] |
| 25 | 2004 | Jiyuan City, Henan Province | 10 | [25] |
| 26 | 2004 | Zhengzhou City, Henan Province | 26 | [26] |
| 27 | 2005 | Lishui City, Zhejiang Province | 54 | [27] |
| 28 | 2005 | 65651 Army Hospital | 2 | [28] |
| 29 | 2005 | Yixian County, Liaoning Province | 2 | [29] |
| 30 | 2006 | Guangzhou City, Guangdong Province | 18 | [30] |
| 31 | 2009 | Xi'an, Shanxi province | 36 | [31] |
| 32 | 2009 | Zhaoyang City, Liaoning Province | 9 | [32] |
| 33 | 2010 | Fushun, Liaoning province | 19 | [33] |
|  |  |  | A total of 833 |  |

**References**

[1] He MF. Investigation of 42 cases of food poisoning caused by eating fresh *H. citrina* [J]. Modern Medical Journal, 1978, (2): 92-92.

[2] Sun DM. Investigation and analysis of 94 cases of edible fresh *H. citrina* poisoning [J]. People's Military Surgeon,1981(07): 28.

[3] Liu GS. A report of 27 cases of food poisoning caused by fresh *H. citrina* [J]. Chinese Journal of School Health, 1989, 10(2): 53-53.

[4] Wang QF. Epidemiological investigation of food poisoning caused by eating fresh *H. citrina* [J]. People's Military Surgeon, 1995, (9): 23-24.

[5] Wang LF, Wang MW, Xu K. Investigation of poisoning by eating fresh *H. citrina* [J]. Preventive Medicine, 1995, 7(6): 31-31.

[6] Yuan R, Sun XW. An investigation report of food poisoning caused by eating fresh *H. citrina* [J]. Preventive Medicine Tribune, 1997, 3(1): 60-61.

[7] Wang PS, Wu B. An investigation of collective food poisoning caused by eating fresh *H. citrina* [J]. South China Journal of Preventive Medicine, 1997(03): 43-44.

[8] Wang MY, Li J. Fresh *H. citrina* poisoning [J]. Chinese Journal of Medicine, 1999, 34(10): 28-28.

[9] Qi GC, Wei YL. Analysis of a case of food poisoning caused by eating fresh *H. citrina* [J]. Preventive Medicine Tribune, 2001, 1: 2.

[10] Lv YA, Su Y, Yu B. One case of poisoning caused by fresh *H. citrina* [J]. Occupation and Health, 2000, 16(10): 64-64.

[11] Song TX, Wang JJ. An investigation of food poisoning caused by eating fresh *H. citrina* [J]. Chinese Journal of School Health, 2000(05): 412.

[12] Wang JF, Wang HS, Xu LF. Investigation report on a collective poisoning caused by students eating fresh *H. citrina* [J]. Chinese Journal of School Health, 2000(04): 274.

[13] Wang XZ, Li SZ. Three cases of fresh *H. citrina* poisoning [J]. Qingdao Medical Journal, 2001, 33(3): 227-227.

[14] Pan QH, He JK. An investigation of food poisoning caused by fresh *H. citrina* [J]. Strait Journal of Preventive Medicine, 2001, 7(6): 60-60.

[15] Jiang CX, Peng H, Qin KB. An investigation report of food poisoning caused by eating *H. citrina* [J]. Anhui Journal of Preventive Medicine, 2001, 17(4): 309-309.

[16] Wu XM. A food poisoning caused by eating fresh *H. citrina* [J]. Preventive Medicine Tribune, 2001(04): 425.

[17] Cheng HJ, Guo L. Two cases of fresh *H. citrina* poisoning [J]. Medical & Pharmaceutical Journal of Chinese People's Liberation Army, 2002, 14(4): 301.

[18] Han CZ. Clinical analysis of two cases of fresh *H. citrina* poisoning [J]. Chinese Community Doctors, 2002, 18(23): 47-47

[19] Qin HX, Li MQ, Chen B. Food poisoning caused by eating fresh *H. citrina* together [J]. Applied Preventive Medicine, 2004, 10(1): 41-41.

[20] Peng LZ. Clinical analysis of 34 cases of fresh *H. citrina* poisoning [J]. Clinical Focus, 2005, 20(12): 675-675

[21] Li EW, Chen WW. The administrative sanitation punishment of a fresh *H. citrina* food poisoning case [J]. Journal of Medical Pest Control, 2006, 22(2): 124-125.

[22] Zhang KY. Plant, food, poison[J]. Family Medicine, 2004(1)：46-46.

[23] Liu JJ, Liu XM, Sun P. Investigation of a food poisoning accident caused by migrant workers on construction site eating fresh *H. citrina* [J]. Occupation and Health, 2005, 21(8): 1174-1175.

[24] Wang SY, Yang YK, Xu QH. An investigation of food poisoning caused by eating fresh *H. citrina* [J]. Chinese Journal of Public Health, 2005, 21(6): 646-646.

[25] Jia HH. An investigation of food poisoning caused by *H. citrina* [J]. Occupation and Health, 2005, 21(2): 188-188.

[26] Chen H, Dong ML. Analysis of 26 cases of fresh *H. citrina* poisoning [J]. Medical Journal of Chinese People's Health, 2008, 20(19): 2252-2252.

[27] Xu WJ, Chen HJ, Song C. Investigation of an outbreak of fresh *H. citrina* poisoning [C].

Volume 7 of The Second Zhejiang Midwest Science and Technology Forum, 2005, 161-163.

[28] Liu W, Xiang GD, Zhou WC. Two cases of fresh *H. citrina* poisoning [J]. Shenyang Army Medical, 2006, 19(3): 187-187.

[29] Liu W. Two cases of colchicine poisoning caused by fresh *H. citrina* [J]. People's Military Surgeon, 2006, 49(11): 676-677.

[30] Peng SL. Clinical analysis of 18 cases of fresh *H. citrina* poisoning [J]. National Medical Frontiers of China, 2007, 2(10): 98-98.

[31] Jiang XH. Experience of emergency treatment of 36 cases of fresh *H. citrina* poisoning [J]. Jilin Medical Journal, 2010, 31(24): 4136-4137.

[32] Guo CL, Nie JS. Investigation of a food poisoning caused by eating fresh *H. citrina* [J]. The Chinese and Foreign Health Abstract, 2011, 08(36): 256-257.

[33] Wu HM. Investigation report on a food poisoning accident in an enterprise canteen [J]. Chinese Journal of Urban and Rural Enterprise Hygiene, 2013, (3): 100-101.

Fig. S1 Comparison of gene features between *H. citrina* and the six other plant species (*Solanum tuberosum*, *Oryza sativa*, *Arabidopsis thaliana*, *Lonicera japonica*, *Asparagus setaceus*, and *Macleaya cordata*). Gene features include gene length, CDS length, exon length and intron length.


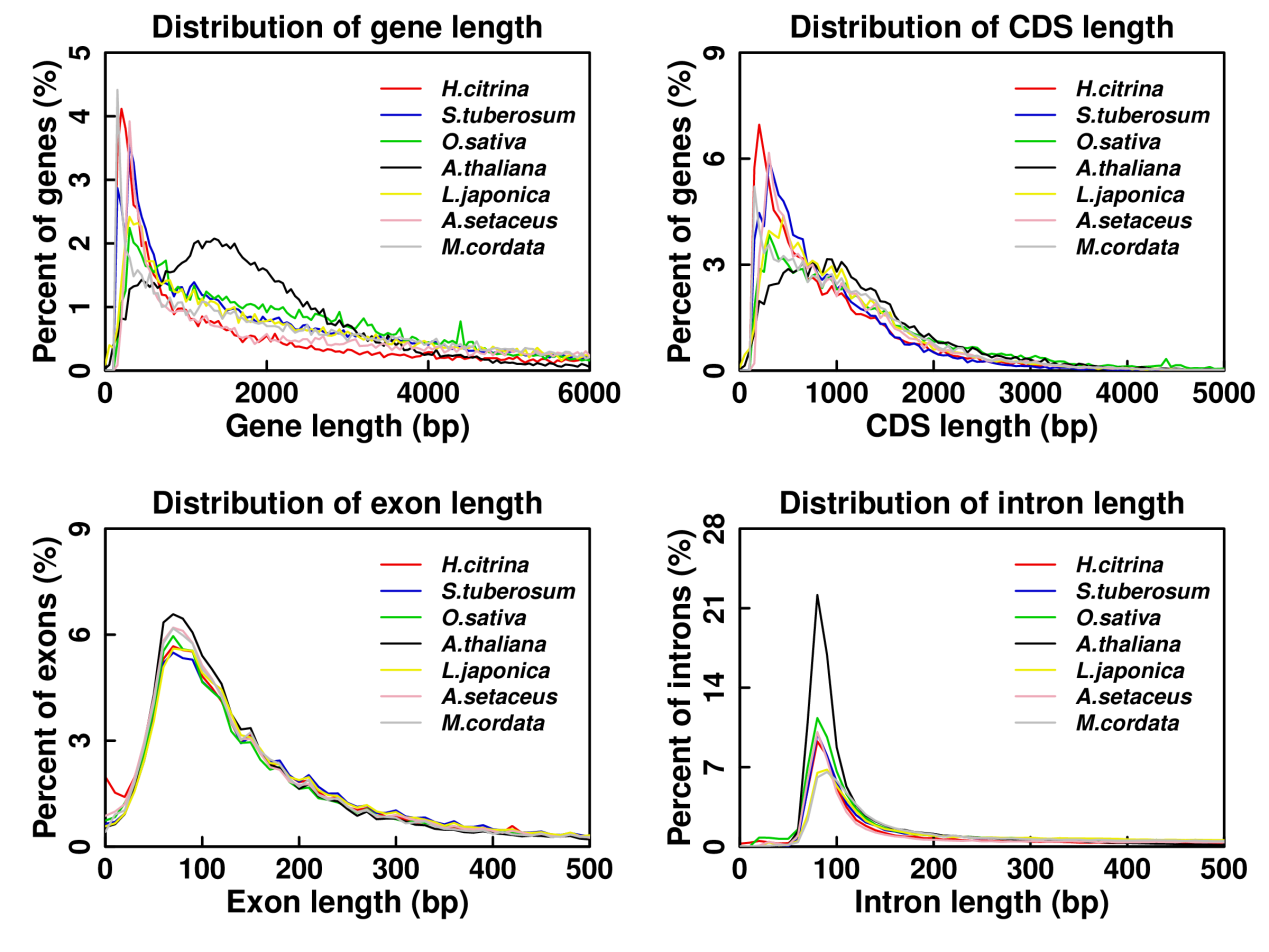


Fig. S2 Characterization of Cp-1. ***a***, EIC of theoretical *m/z* values (*m/z* 182.0812) of Cp-1 in the TICs of *G. superba*, *C. autumnale*, and *H.citrina*, respectively. ***b***, MS/MS spectra of observed parent ions at *m/z* 182.0803, 182.0817, and 182.0798 from the TICs of *G. superba*, *C. autumnale*, and *H.citrina*, respectively. ***c***, The tabulated list and putative structures for the fragment ions from the MS/MS analysis of the mother ion at *m/z* 182.0803.

Fig. S3 Characterization of Cp-2. ***a***, EIC of theoretical *m/z* values (*m/z* 154.0863) of Cp-2 in the TICs of *G. superba*, *C. autumnale*, and *H.citrina*, respectively. ***b***, MS/MS spectra of observed parent ions at *m/z* 154.0864 and 154.0869 from the TICs of *G. superba* and *C. autumnale* respectively. ***c***, The tabulated list and putative structures for the fragment ions from the MS/MS analysis of the mother ion at *m/z* 154.0864.

Fig. S4 Characterization of Cp-3. ***a***, EIC of theoretical *m/z* values (*m/z* 166.0863) of Cp-3 in the TICs of *G. superba*, *C. autumnale*, and *H.citrina*, respectively. ***b***, MS/MS spectra of observed parent ions at *m/z* 166.0856, 166.0852, and 166.0852 from the TICs of *G. superba*, *C. autumnale*, and *H.citrina*, respectively. ***c***, The tabulated list and putative structures for the fragment ions from the MS/MS analysis of the mother ion at *m/z* 166.0856.

Fig. S5 Characterization of Cp-4. ***a***, EIC of theoretical *m/z* values (*m/z* 151.0754) of Cp-4 in the TICs of *G. superba*, *C. autumnale*, and *H.citrina*, respectively. ***b***, MS/MS spectra of observed parent ions at *m/z* 151.0754 and 151.0737 from the TICs of *G. superba* and *C. autumnale* respectively. ***c***, The tabulated list and putative structures for the fragment ions from the MS/MS analysis of the mother ion at *m/z* 151.0754.

Fig. S6 Characterization of Cp-5. ***a***, EIC of theoretical *m/z* values (*m/z* 286.1438) of Cp-5 in the TICs of *G. superba*, *C. autumnale*, and *H.citrina*, respectively. ***b***, MS/MS spectra of observed parent ions at *m/z* 286.1436 and 286.1423 from the TICs of *G. superba* and *C. autumnale* respectively. ***c***, The tabulated list and putative structures for the fragment ions from the MS/MS analysis of the mother ion at *m/z* 286.1436.

Fig. S7 Characterization of Cp-6. ***a***, EIC of theoretical *m/z* values (*m/z* 300.1594) of Cp-6 in the TICs of *G. superba*, *C. autumnale*, and *H.citrina*, respectively. ***b***, MS/MS spectra of observed parent ions at *m/z* 300.1597 and 300.1599 from the TICs of *G. superba* and *C. autumnale* respectively. ***c***, The tabulated list and putative structures for the fragment ions from the MS/MS analysis of the mother ion at *m/z* 300.1597.

Fig. S8 Characterization of Cp-7. ***a***, EIC of theoretical *m/z* values (*m/z* 314.1751) of Cp-7 in the TICs of *G. superba*, *C. autumnale*, and *H.citrina*, respectively. ***b***, MS/MS spectra of observed parent ions at *m/z* 314.1734 and 314.1765 from the TICs of *G. superba* and *C. autumnale* respectively. ***c***, The tabulated list and putative structures for the fragment ions from the MS/MS analysis of the mother ion at *m/z* 314.1565.

Fig. S9 Characterization of Cp-8. ***a***, EIC of theoretical *m/z* values (*m/z* 330.1700) of Cp-8 in the TICs of *G. superba*, *C. autumnale*, and *H.citrina*, respectively. ***b***, MS/MS spectra of observed parent ions at *m/z* 330.1666 and 330.1718 from the TICs of *G. superba* and *C. autumnale* respectively. ***c***, The tabulated list and putative structures for the fragment ions from the MS/MS analysis of the mother ion at *m/z* 330.1718.

Fig. S10 Characterization of Cp-9. ***a***, EIC of theoretical *m/z* values (*m/z* 360.1805) of Cp-9 in the TICs of *G. superba*, *C. autumnale*, and *H.citrina*, respectively. ***b***, MS/MS spectra of observed parent ions at *m/z* 360.1812 and 360.1788 from the TICs of *G. superba* and *C. autumnale* respectively. ***c***, The tabulated list and putative structures for the fragment ions from the MS/MS analysis of the mother ion at *m/z* 360.1812.

Fig. S11 Characterization of Cp-10. ***a***, EIC of theoretical *m/z* values (*m/z* 374.1962) of Cp-10 in the TICs of *G. superba*, *C. autumnale*, and *H.citrina*, respectively. ***b***, MS/MS spectra of observed parent ions at *m/z* 374.1952 and 374.1934 from the TICs of *G. superba* and *C. autumnale* respectively. ***c***, The tabulated list and putative structures for the fragment ions from the MS/MS analysis of the mother ion at *m/z* 374.1952.

Fig. S12 Characterization of Cp-11. ***a***, EIC of theoretical *m/z* values (*m/z* 372.1805) of Cp-11 in the TICs of *G. superba*, *C. autumnale*, and *H.citrina*, respectively. ***b***, MS/MS spectra of observed parent ions at *m/z* 372.1805 and 372.1796 from the TICs of *G. superba* and *C. autumnale* respectively. ***c***, The tabulated list and putative structures for the fragment ions from the MS/MS analysis of the mother ion at *m/z* 372.1805.

Fig. S13 Characterization of Cp-12. ***a***, EIC of theoretical *m/z* values (*m/z* 386.1962) of Cp-12 in the TICs of *G. superba*, *C. autumnale*, and *H.citrina*, respectively. ***b***, MS/MS spectra of observed parent ions at *m/z* 386.1959 and 386.1957 from the TICs of *G. superba* and *C. autumnale* respectively. ***c***, The tabulated list and putative structures for the fragment ions from the MS/MS analysis of the mother ion at *m/z* 386.1959.

Fig. S14 Characterization of Cp-13. ***a***, EIC of theoretical *m/z* values (*m/z* 400.1755) of Cp-13 in the TICs of *G. superba*, *C. autumnale*, and *H.citrina*, respectively. ***b***, MS/MS spectra of observed parent ions at *m/z* 400.1761 and 400.1741 from the TICs of *G. superba* and *C. autumnale* respectively. ***c***, The tabulated list and putative structures for the fragment ions from the MS/MS analysis of the mother ion at *m/z* 400.1761.

Fig. S15 Characterization of Cp-14. ***a***, EIC of the theoretical *m/z* value (372.1805, [M+H]^+^) of Cp-14 in the TICs of *G. superba*, *C. autumnale*, *H. citrina*, and the standard. ***b***, MS/MS spectra of the parent ions at *m/z* 372.1822 and 372.1805 from the TICs of *G. superba* and *C. autumnale*, respectively, compared to the demecolcine standard (Cp-14, *m/z* 372.1809). ***c***, The tabulated list and putative structures for the fragment ions from the MS/MS analysis of the mother ion at *m/z* 372.1805.

Fig. S16 Characterization of Cp-15. ***a***, EIC of the theoretical *m/z* value (358.1649, [M+H]^+^) of Cp-15 in the TICs of *G. superba*, *C. autumnale*, *H. citrina*, and the standard. ***b***, MS/MS spectra of the parent ions at *m/z* 358.1660 and 358.1627 from the TICs of *G. superba* and *C. autumnale*, respectively, compared to the deacetylcolchicine standard (Cp-15, *m/z* 358.1651). ***c***, The tabulated list and putative structures for the fragment ions from the MS/MS analysis of the mother ion at *m/z* 358.1660.

Fig. S17 K-mer depth and number frequency distribution diagram.


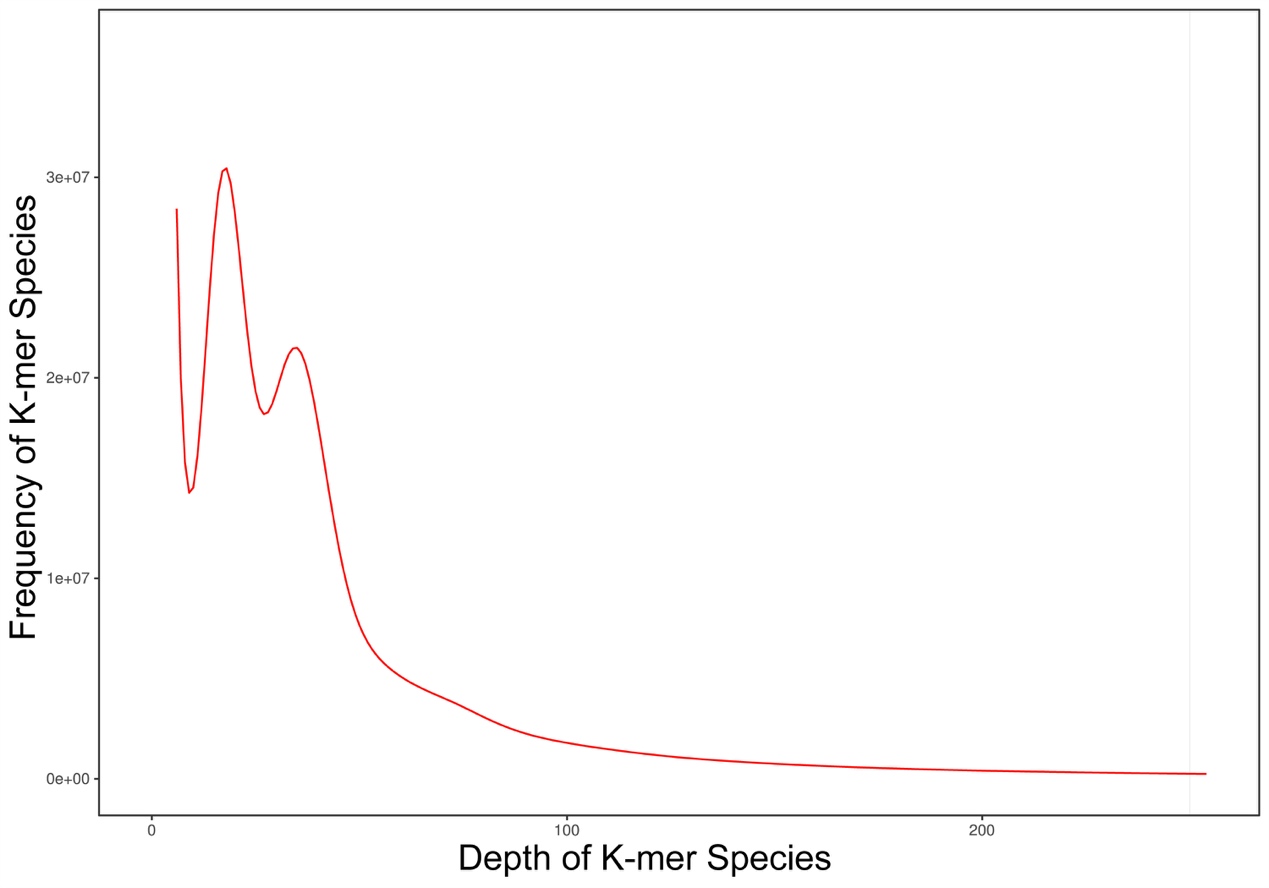


Fig. S18 Contig GC content and sequencing depth distribution density map.


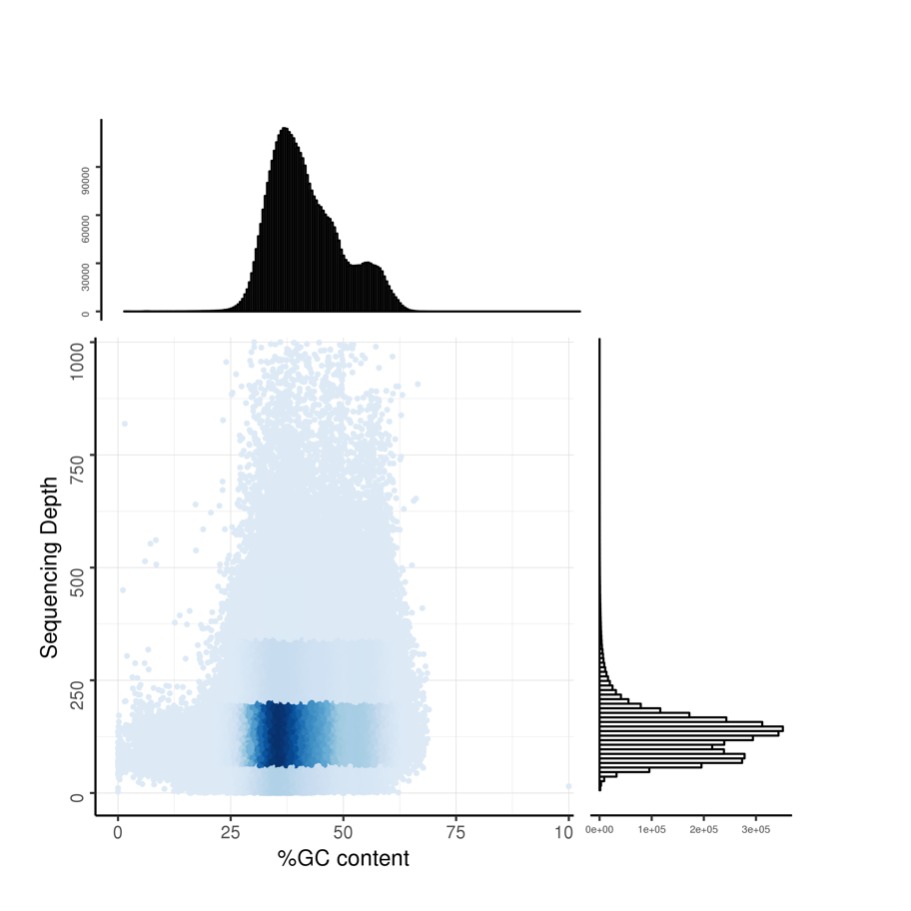

Supplement: Supplementary file 1 — Supporting Material [file 41438_2021_539_MOESM1_ESM.doc]
